# Febrile Intermittent Diarrhœa in Camels

**Published:** 1886-07

**Authors:** Richard W. Burke

**Affiliations:** Army Veterinary Department, Cawnpore, India


THE JOURNAL

OF

COMPARATIVE MEDICINE AND SURGERY.

VOL. VII. JULY, 1886. No. 3

### ORIGINAL COMMUNICATIONS.

### ART. XII.—FEBRILE INTERMITTENT DIARRHŒA IN CAMELS.

BY RICHARD W. BURKE, M.R.C.V.S.,
*Army Veterinary Department, Cawnpore, India.*

The importance of obtaining reliable information regarding diseases of the Camel in India, is so generally recognized as to call for little comment. It is sad to reflect that the frequent mortality among Camels in all large transport stations throughout the country is so high—a circumstance attributable in a very large measure to the indifference or culpable neglect shown in the care of transport animals, and the paucity of Veterinary Surgeons to effectually cope with outbreaks of contagious disease under similar circumstances.

We have only recently brought to a conclusion our endeavor to suppress an outbreak of Anthrax in Camels, an account of which appeared in the *Veterinary Journal*, for January of this year, and we wish briefly to describe an Enzooty of Febrile Intermittent Diarrhœa in those animals. We find diarrhœa due to so-called "Abdominal form of Anthrax," observed by many veterinarians, not of that nature. From the study of clinical facts, we must draw the conclusion that the disease is caused by the *fungus malaria*, and is nothing else but febrile intermittent diarrhœa. The animals in question were employed in removing dung heaps for the space of about two months, when an enzooty of intermittent diarrhœa, very fatal in its character, was seen to follow, with such marked pertinacity, that a Veterinary Surgeon was consulted.

Some have maintained that true Anthrax may thus be produced, and to this they ascribe many of the remarkable successes obtained through the use of carbolic acid.* The good effects once hoped from the internal administration of carbolic acid are seen not to be obtainable, or only to a very limited extent, and in a small percentage of cases. Except in a few cases mentioned, carbolic acid has little influence on the diarrhœa of camels, which is intermittent in character, and nearly always proves fatal in its effects. The temperature at the height of the fever was in the majority of cases, 105° F., and fell in the remissions, having a periodic tendency, to 99°F. If acute diarrhœa cannot be checked, the result is increased activity, of the bowels, inanition and rapid death. Better results were obtained when sulphuric acid dil. with opium, was used instead of the carbolic, and chloroform † when the fœces contained much mucous shreds or blood ; but I would urge a trial of the action of acid sulphuric on a larger scale and in a greater number of cases, and a more extended clinical observation of the results obtained, before the precise significance of the treatment is recognized.

In treating this disease, the diarrhœa must be checked, as well as the system supported, as the strength of the patient must be sustained to a point which shall overcome the weakening influence of hyperaction of the bowels.

The only pathological condition met with on post-mortem examinations was congestion of the mucosa of the intestinal tract, more or less throughout its whole extent, with patchy elevations due to gelatinous exudation, containing the *fungus malaria*—a filamentous many-jointed organism, which grows readily in blood-serum, urine, gelatin, ordinary gum, rice—konjee and other media. Before cultivation they often appear

*Vratch, No. 10, 1885, p. 146; et Vratch, No. 27, 1885, p. 428.

† Journal de Med. et de Chirurgie Pratiques, April, 1885, only as simple small rods, recognizable only under the higher powers of the microscope. Mounted fresh on glass plate and examined under a 1-12 obj. (Benèche), they could be easily recognized.

The spleen and other organs did not appear to be the seat of special disease, beyond slight congestion noticed in a few cases.

Whilst many of the fungi may be eaten with impunity, others contain toxic principles which are of interest clinically and pathologically. Agaricin, the alkaloid of the agaricus laricis or larch agaric, and muscarin, which is derived from the agaricus muscarinus or fly agaric have long been known. More recently several other fungi have been examined and their toxic action determined.

The Helvella esculenta is a mushroom-like fungus which is usually described among the edible fungi, but in several cases has produced serious symptoms of poisoning. Vomiting, diarrhœa, abdominal pain, icterus, convulsions, etc., have followed its use as food, these symptoms, on several occasions, terminating in death. Bostroem * pointed out that besides diarrhœa and vomiting, it causes in dogs hœmoglobinuria, followed sometimes by complete anuria; consciousness is lost, the reflexes disappear, but the breathing is frequent and the heart beats with vigor. Icterus, due to the dissolution of the red corpuscles, is frequently present, and dyspnœa likewise occur. Ponfick † has recorded similar symptoms after the exhibition of this fungus. Boehn and Kulz ‡ confirm the physiological and pathological effects noted by the above observer.

The Boletus luridus is a fungus found chiefly in woods; it is of a dark brown color, and often of considerable size,

The Amanita Pantherina is closely allied to the Amaniat muscaria, from which it differs, indeed, chiefly in the color of its cap, which is brown, whilst the fly agaric is red.

The action of Cholin, often called Neurin, on man, has no been determined. It has been found in the Helvella esculenta, Boletus iuridus and Amanita pantherina, as a white crystalline substance. In frogs it causes general paralysis, with twitching

* Deut. Arch. fur. Kl. Med,, 1882, p. 209.

† Virchow's Archiv., vol. 88.

‡ Archiv. f. exp. Path. und Pharm., 1885, vol. xix, pp. 403-414.

and trembling of the muscles; in rabbits it caused salivation, but large doses produced no paralysis or other nervous symptoms; in cats, besides salivation, the administration of Cholin was followed by great weakness in the hinder extremities and general paralysis.

Glause and Juchsinger * have examined the action of Cholin, muscarin and other bases; they affirm the muscarin action of all, and from their experiments conclude that muscarin paralyses especially the heart muscle, and that this effect is antagonized by atropin.

* Fortschritte d. Med., 1884, No. 8, p. 276.

